# Paralog-based synthetic lethality: rationales and applications

**DOI:** 10.3389/fonc.2023.1168143

**Published:** 2023-06-07

**Authors:** Yucui Xin, Yingsheng Zhang

**Affiliations:** The Innovation Center, Beijing StoneWise Technology Co Ltd., Beijing, China

**Keywords:** cancer therapy, synthetic lethality, paralog, clinical prospect, mechanism

## Abstract

Tumor cells can result from gene mutations and over-expression. Synthetic lethality (SL) offers a desirable setting where cancer cells bearing one mutated gene of an SL gene pair can be specifically targeted by disrupting the function of the other genes, while leaving wide-type normal cells unharmed. Paralogs, a set of homologous genes that have diverged from each other as a consequence of gene duplication, make the concept of SL feasible as the loss of one gene does not affect the cell’s survival. Furthermore, homozygous loss of paralogs in tumor cells is more frequent than singletons, making them ideal SL targets. Although high-throughput CRISPR-Cas9 screenings have uncovered numerous paralog-based SL pairs, the unclear mechanisms of targeting these gene pairs and the difficulty in finding specific inhibitors that exclusively target a single but not both paralogs hinder further clinical development. Here, we review the potential mechanisms of paralog-based SL given their function and genetic combination, and discuss the challenge and application prospects of paralog-based SL in cancer therapeutic discovery.

## 1 Introduction

Gene duplication is a common phenomenon in cellular evolution, serving as a primary method for the creation of novel genes (1). Paralogs are genes that originate from gene duplication events (2). These duplicated genes can be retained through mechanisms such as “neo-functionalization”, “sub-functionalization”, “dosage amplification” and “back-up compensation,” or become non-functional through a process called “non-functionalization” (1). Despite various evolutionary patterns, many paralogs retain a degree of functional redundancy, which may lead to back-up compensation and confer genetic robustness and adaptive advantages (3–5). For example, it has been observed in both budding yeast and human cells that the loss of function of paralogs is less detrimental than that of singletons (3, 6). The compensation mechanism of paralogs shows greater phenotypic plasticity in various environmental stress conditions. The redundant copies can be free from the constraints of natural selection and obtain “forbidden mutations” that allow the development of new or more specialized functions (1). An example of this is the MSN2-MSN4 paralogs in yeast. Following a gene duplication event, the expression of MSN2 became highly stable and less responsive to environmental changes (low-noise-based expression), while the expression of MSN4 became more dynamic and had random variations (noise). This provided yeast with the opportunity to evolve phenotype-adaptive expression tuning (7).

**Figure 1:**
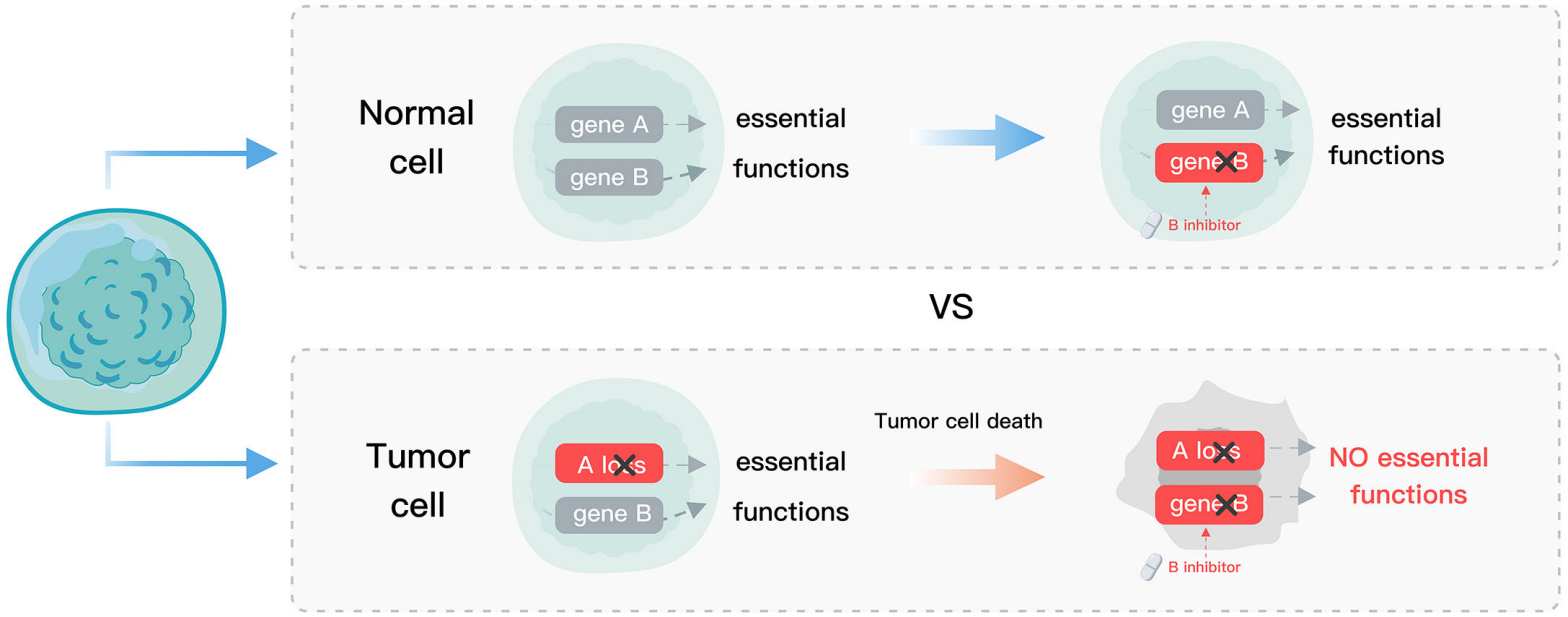
Schematic diagram of SL. Normal cells, devoid of gene A mutations, exhibit tolerance to gene B suppression, whereas tumor cells with gene A loss undergo gene B suppression, leading to cell death.

Homozygous paralog loss occurs more frequently than singletons in cancer (8), suggesting that tumor cells that can tolerate gene loss were selected. However, this also makes the tumors vulnerable as only one of the paralogs is retained (8). Synthetic lethality (SL), a promising strategy for precision cancer medicine (9), uses drugs to target the genes with complementary functions in a tumor that has acquired a certain mutation. This approach results in the complete disruption of the targeted genes’ functions and a subsequent defect in tumor cell viability, while normal cells with the un-mutated gene remain unaffected (**Figure 1**). The concept of SL overcomes some limitations faced by traditional precision medicine such as a small number of targets and undruggable genes (10). Poly (ADP-ribose) polymerase (PARP) inhibitors developed based on the SL interaction between PARP and BRCA have provided substantial clinical benefit to patients (11–16), and demonstrated the feasibility of SL for precision oncology. The application of SL in precision cancer medicine is facilitated by the presence of many paralog buffer systems in cells and the specific mutations that occur in tumor cells. Therefore, the paralog vulnerability of tumor cells can be leveraged to specifically target the functionally compensating genes corresponding to the mutated paralog genes, thus achieving the goal of selectively eliminating tumor cells.

Approximately 70.5% of the 19,430 protein-coding genes in the human genome have one or more paralogs (17). Despite the extensive screening of over 700 cancer cell lines using genome-wide CRISPR-Cas9 and RNA interference (RNAi) libraries (DepMap portal, https://depmap.org/portal/), new targets related to the most common genetic drivers have not yet been discovered (18). This is likely contributed by the functional redundancies among paralogous genes, which can mask the dependencies to single-gene perturbation. Thus, paralogs with functional buffering are not only promising candidates for SL, but abnormalities in paralog members can also serve as crucial biomarkers for identifying context-specific SL targets. This review will systematically explore paralog-based SL interactions according to their various functional categories and practical applications. Additionally, we will propose a new paralog-based SL genetic combination and its underlying mechanism. Finally, we will discuss the challenges and prospects of paralog-based SL in drug discovery and development.

## 2 Functional categories of paralog-based SL

The effectiveness of paralog-based SL often relies on whether the complex and the pathway they belong to have essential functions (10). Mapping the functions of paralog-based SL pairs can serve as a reliable starting point for further research. Current confirmed essential cellular functions of SL pairs include gene expression regulation, the cell cycle, DNA damage repair, energy metabolism and material transport. We summarized in **Table 1** paralog-based SL interactions that have been validated using low-throughput experiments.

**Table 1:** SL interactions within the paralog family.

Paralog1	Paralog 2	Description of encoding protein	Cancer/Cell types	Prevalence in cancer	References
ARID1A	ARID1B	Subunits of the SWI/SNF complex	ARID1A- mutant ovarian cancer cells	ARID1A-deficient	(19)
SMARCA2	SMARCA4		Lung cancer	SMARCA4 mutation	(20–22)
CREBBP	EP300	Protein-Lysine Acetyltransferase	Lung cancer, Hematopoietic cancer, Bladder Cancer and Diffuse large B-cell lymphoma	CREBBP-deficient	(23–25)
HDAC1	HDAC2	Histone deacetylases	Neuroblastoma and multiple myeloma	HDAC1 hemizygous deletion	(26)
STAG1	STAG2	A cohesin subunit	Acute myeloid leukemia, Ewing sarcoma and bladder cancers	STAG2 mutation	(27–30)
CSTF2	CSTF2T	Cleavage stimulation factor subunit 2 and 2Tau	Lung adenocarcinoma and melanoma cell lines	CSTF2T-deficient	(31)
MAGOH	MAGOHB	Core members of the splicing-dependent exon junction complex	Gastric Cancer		(32)
VRK1	VRK2	Nuclear serine-threonine kinase	Glioblastoma, gliomas and neuroblastomas	VRK2 promoter methylation	(33, 34)
ENO1	ENO2	The glycolytic gene enolase	Glioblastoma	ENO1 deleted	(35)
ME2	ME3	Mitochondrial malic enzyme	Pancreatic ductal adenocarcinoma	ME2 deletion	(36)
VPS4A	VPS4B	ATPases of the endosomal sorting complex (ESCRT)	Colorectal cancer, Rhabdomyosarcoma and pancreatic ductal adenocarcinoma	VPS4A or VPS4B deletion	(37, 38)
NXT1	NXT2	Nuclear export factors	Neuroblastoma	Low NXT2 expression	(39)
UBB	UBC	Ubiquitin	High-grade serous ovarian cancer, uterine carcinosarcoma and endometrial carcinoma	Transcriptional repression of ubiquitin B	(40)
DDX3X	DDX3Y	DEAD-Box Helicase 3	Lymphoma	loss-of-function DDX3X mutations, Loss of Y	(41, 42)
SMARCC1	SMARCC2	Subunits of the SWI/SNF complex	SK-MES-1	Harboring an SNP in SMARCC1	(43)
RBM26	RBM27	RNA-binding proteins	HAP1 and RPE1		(44)
SREBF1	SREBF2	Sterol regulatory element binding transcription factor	HAP1		(45)
DDX5	DDX17	DEAD-Box Helicase	HAP1		(46)
PTDSS1	PTDSS2	Phosphatidylserine synthetase	HCT116	PTDSS2 loss	(47)
SLC16A1	SLC16A3	lactate transporter monocarboxylate transporter	HAP1, K-562 and BV-173	SLC16A3 low expression	(44, 48)
LDHA	LDHB	Lactate Dehydrogenase	HAP1		(44)
RPP25	RPP25L	A component of RNase P and RNase MRP ribonuclease complexes	U-2OS and KYSE-150		(42)
DNAJC15	DNAJC19	Negative regulator of the mitochondrial respiratory chain	CAL-12T, NCI-H1915 and NCI-H1975	DNAJC15 promoter methylation	
EIF1AX	EIF1AY	Eukaryotic Translation Initiation Factor 1A	KNS-42	LOY	
ZFX	ZFY	Probable transcriptional activator			
FAM50A	FAM50B	Probably involved in the regulation of pre-mRNA splicing	ESS-1 and NCI-H1915	FAM50B promoter methylation	
ASF1A	ASF1B	Members of the H3/H4 family of histone chaperone proteins	HAP1	ASF1A deletion	(49)
COPS7A	COPS7B	Components of the COP9 signalosome		COPS7B deletion	

### 2.1 Regulation of gene expression

Epigenetic factors play a crucial role in the regulation of transcription and expression of many fundamental genes, and have demonstrated potential as therapeutic targets in the field of SL. Mutations in genes encoding subunits of the SWI/SNF chromatin remodeling complex are commonly found in over 20% of known human cancers and are thought to promote tumorigenesis by disrupting transcriptional homeostasis (50, 51). The ARID1A-ARID1B, SMARCA2-SMARCA4, and SMARCC1-SMARCC2, subunits of the SWI/SNF complex, have been proven to have SL interactions (19, 20, 22, 43, 52). CREBBP-EP300 can increase the accessibility of gene transcription (21, 53). Co-deletion of them triggers SL in certain cancers (23–25). Histone deacetylases (HDACs) alter chromatin structures to modulate transcription levels of nearby genes and lead to the down-regulation of cell cycle regulators and tumor suppressors (54). Loss of HDAC2 produces SL effects in HDAC1 hemizygous deletion cells (26). In addition to epigenetic factors, the cohesin complex regulates gene expression by forming a DNA ring, and its members STAG1 and STAG2 have a strong SL interaction (27, 30, 55–57). The RNA-binding proteins RBM26 and RBM27 play a critical role in mRNA processing, and their simultaneous depletion leads to a synergistic reduction in cell viability (44, 58). Sterol regulatory element binding transcription factors SREBF1 and SREBF2 show a strong reciprocal SL interaction (45). RPP25 is a component of the Th/To complex that processes a variety of RNAs (59). Its low expression makes cells sensitive to RPP25L loss (42, 60). DDX5 and DDX17 are members of the DEAD box family, primarily involved in transcription and splicing processes. They have been identified as an SL gene pair through high-throughput screening (46). CSTF2 and CSTF2T are involved in the mRNA cleavage and polyadenylation (61). The knockout of CSTF2 deletes tumor cells with homozygous CSTF2T deletions (31, 42). The core members of the exon junction complex, MAGOH and MAGOHB, are essential for mRNA processing, and their combination knockdown results in lethality (32, 62).

### 2.2 Cell-cycle and DNA damage repair

PARP inhibitors are currently approved for treating advanced ovarian and breast cancers that are caused by mutations in the BRCA1/2 genes and are used as second-line therapy (63). Successfully leveraging the SL interaction between PARP and BRCA has led to a focus on identifying genes involved in the cell cycle and DNA damage repair as potential SL targets. It is worth noting that genes in this category tend to have multiple functions. For example, in addition to regulating gene expression, the cohesin complex also has a canonical cell-cycle-associated function (28)*.* Co-inactivation of its subunits STAG1 and STAG2 can lead to loss of sister chromatid cohesion and cell death. Mutation in STAG2 also causes replication fork stall and collapse, making corresponding tumor cells more sensitive to certain chemotherapy and inhibitors targeting DNA double-strand break (DSB) repair genes (27, 57, 64). The nuclear serine-threonine kinase VRK1, which plays a role in regulating the cell cycle and DNA damage repair has been found to have an SL interaction with VRK2 (33, 34). Perhaps due to more buffer systems in the cell cycle and DNA repair regulation, the discovery of SL within paralog families in these processes is well below the expectation. However, more SL interactions between paralog members and other genes have been discovered and verified, such as TLK1/TLK2 and PARP (65), SMARCA4 and CDK4/6 (66). In addition, CDK1, CDK2, CDK12, and CDK17 all have been confirmed to have SL interaction with other genes (9, 67). Further insights into the cyclin-dependent kinases-based SL interactions can be obtained by consulting the review by Li et al. (68).

### 2.3 Energy metabolism and material transport

Tumorigenesis is heavily influenced by metabolic responses, as cancer cells have a high bioenergetic demand but also are restricted by limited nutrient availability in the tumor microenvironment (69). The loss of the gene encoding metabolic enzymes in cancer cells leads to a dependence on their paralog or redundant metabolic pathway, resulting in the SL phenomenon. For example, with the loss of enolase ENO1, cancer cells are abnormally sensitive to the repression of its redundant gene ENO2 (35, 70). Selective inhibition of ENO2 by either genetic or pharmacological means can inhibit proliferation and trigger apoptosis in ENO1-deficient glioma cells (71). Similarly, loss of mitochondrial malic enzymes ME3 leads to cell death in ME2-mutated pancreatic ductal adenocarcinoma cells (36). Depletion of phosphatidylserine synthase PTDSS1 specifically suppressed growth in PTDSS2-deficient cancer cell lines (47). L-lactate dehydrogenase LDHA and LDHB are essential for the Warburg effect. Inhibition of both LDHA and LDHB could be therapeutically effective (44, 72). Dual inhibition of lactate transporters SLC16A1 and SLC16A3 (44, 48), or combination of SLC16A1-SLC16A4 with metformin (73) leads to cancer cell death.

SL interactions also occur in the transport process, which is crucial for the transfer of energy and materials. For example, the VPS4A and VPS4B, ATPases of the endosomal sorting complex (ESCRT), a polyprotein complex that plays a vital role in reversing membrane remodeling, have been confirmed as an SL gene pair both *in vitro* and *in vivo* (37, 38, 42). Another transport paralog gene pair NXT1 and NXT2 regulates the export of mRNA from the nucleus and is known to have an SL interaction (39).

### 2.4 Other functions

This category includes functions with fewer reported SL interactions. For instance, the simultaneous inactivation of phosphatase DUSP4 and DUSP6 selectively impairs the growth of cells with mutations in NRAS or BRAF by hyperactivating MAPK signaling (18). BCL2L1 and BCL2L2 are a pair of SL genes that are apoptosis-related paralogs, initially identified through dual-gene knockout screening (74, 75). Inhibiting the ubiquitin UBC in tumors of UBB silencing led to tumor regression (40). DDX3X and DDX3Y are DEAD-box RNA helicase that regulates translation and exhibits SL interaction (42, 76). FAM50A and FAM50B, with an unclear function, also exhibit SL interaction (42, 77).

## 3 Clinical development of SL-targeted drugs

It was first shown in 2005 that SL genes can be a selective and effective target for precision cancer treatment. Ashworth and Helleday demonstrated that inhibition of PARP inhibitors selectively kills BRCA1/2-deficient tumor cells, revealing an SL interaction between PARP and BRAC1/2 (12, 13). More clinical and mechanistic details related to BRCA-deficient tumors can refer to the review article by Patel et al. (63). Since then, multiple PARP inhibitors including olaparib, niraparib, rucaparib, talazoparib have been approved for multiple cancers, and many other SL-targeted drugs have been tested in clinical trials. The SL partners of genes including TP53, KRAS, and MYC have been targeted (10). Inhibitors of ATR, WEE1, CHK1, and mTOR, the SL partners of tumor suppressor gene TP53, all showed efficacy in clinical development (10, 78, 79). There is even a large pool of SL genes in preclinical developments including p38MAPK/MK2, PLK1, PIP4K2B, HK2, PDGFR, and PLA2G16 (80). In addition, the SL partners of oncogene KRAS in clinical development are combinatorial targets such as TBK1-MEK, AKT-MEK, and CDK4-MEK (81), and those of MYC are ATR, AURKB, and CDK9 (9, 82).

Although a pair of paralog genes provide a highly straightforward SL interaction by virtue of their similar functions, it is worth noting that drug development from paralog-based SL is still scarce in clinical trials (ClinicalTrials.gov) and cancer therapies (83). The slow development of these paralog-specific inhibitors might be resulting from the difficulty to balance efficacy and toxicity in the complex environment of a tumor, which requires a highly specific inhibitor to spare the non-target paralog despite the nearly identical gene or protein sequence homologies (84). Similarly, the SL interaction between paralogs is seemingly hampered by the lack of suitably selective inhibitors. However, some promising solutions are under development. For example, protein crystal structures can reveal structural differences that may be exploited to confer paralog selectivity for small-molecule ligands (57). PROTACs (PROteolysis TArgeting Chimeras) are hetero-bifunctional chimeric molecules consisting of one protein-binding ligand, one E3 ubiquitin ligase recruiting moiety, and a linker to connect them. PROTACs can pull a targeted protein to an E3 ubiquitin ligase, leading to their ubiquitination and degradation (85). This approach has shown promise in discriminating between similar paralogous genes and offers some opportunities for therapeutic development. For example, the PROTAC degrader PP-C8 confers specificity for CDK12 over CDK13 (86). VHL-recruiting PROTAC ACBI2 selectively degrades SMARCA2 over SMARCA4 (87). Moreover, Antisense oligonucleotides (ASOs) are short nucleic acid fragments that uniquely hybridize with complementary pre-mRNAs or mRNAs to modulate their functions (88, 89), making them a powerful tool for selectively targeting paralog members. For instance, highly selective ASOs have been developed for HK2 inhibition (90) and TYK2 inhibition (91), without affecting the expression level of other paralogs. In addition, for paralog members such as DDX3Y, whose abnormal expression can be specifically recognized by immune cells in leukemic stem cells, immune treatment is a possibility (92). Although selective targeting paralog-based SL interaction for tumor cells has shown a nice clinical value, it is worth noting to consider the protein expression of its paralog in other normal tissues. For example, ENO1 is the only isoform expressed in red blood cells, so pan-enolase inhibition can lead to anemia (71). This suggests that determining the expression subtypes in normal tissues is imperative to prevent damage to certain normal cells.

Sex paralogs provide a new opportunity for male tumors. Some paralogous genes are located between sex chromosomes. The prevalent loss of chromosome Y in male cancer patients makes depletion of the chrX-encoded paralog attractive as a therapeutic strategy. For example, chrX- and chrY-encoded paralogs including DDX3X-DDX3Y, ZFX-ZFY, and EIF1AX-EIF1AY have been proven to buffer for each other’s loss and dependent on chrX-encoded paralog in cancers with loss of chrY (42). Accumulating evidence suggests that targeting the DDX3X-DDX3Y paralogs may be an effective treatment strategy (41, 42, 76, 93). Specifically, inhibiting DDX3X in male patients with loss of chromosome Y, or targeting DDX3Y in male tumors with co-occurring DDX3X and MYC mutations, could hold promise for eliminating cancerous cells. For example, male lymphomas rely on functional compensation by DDX3Y for rescue, and inhibitors blocking DDX3Y can eliminate cancerous B cells (41). Moreover, since DDX3Y protein is not expressed in normal adult cells, the toxicity of therapeutic DDX3Y inhibition would be low. Although some inhibitors have shown some anti-tumor effects, the development of a specific inhibitor is still challenging and requires a deeper understanding of underlying mechanisms.

## 4 Genetic combination and mechanism of paralog-based SL

**Figure 2:**
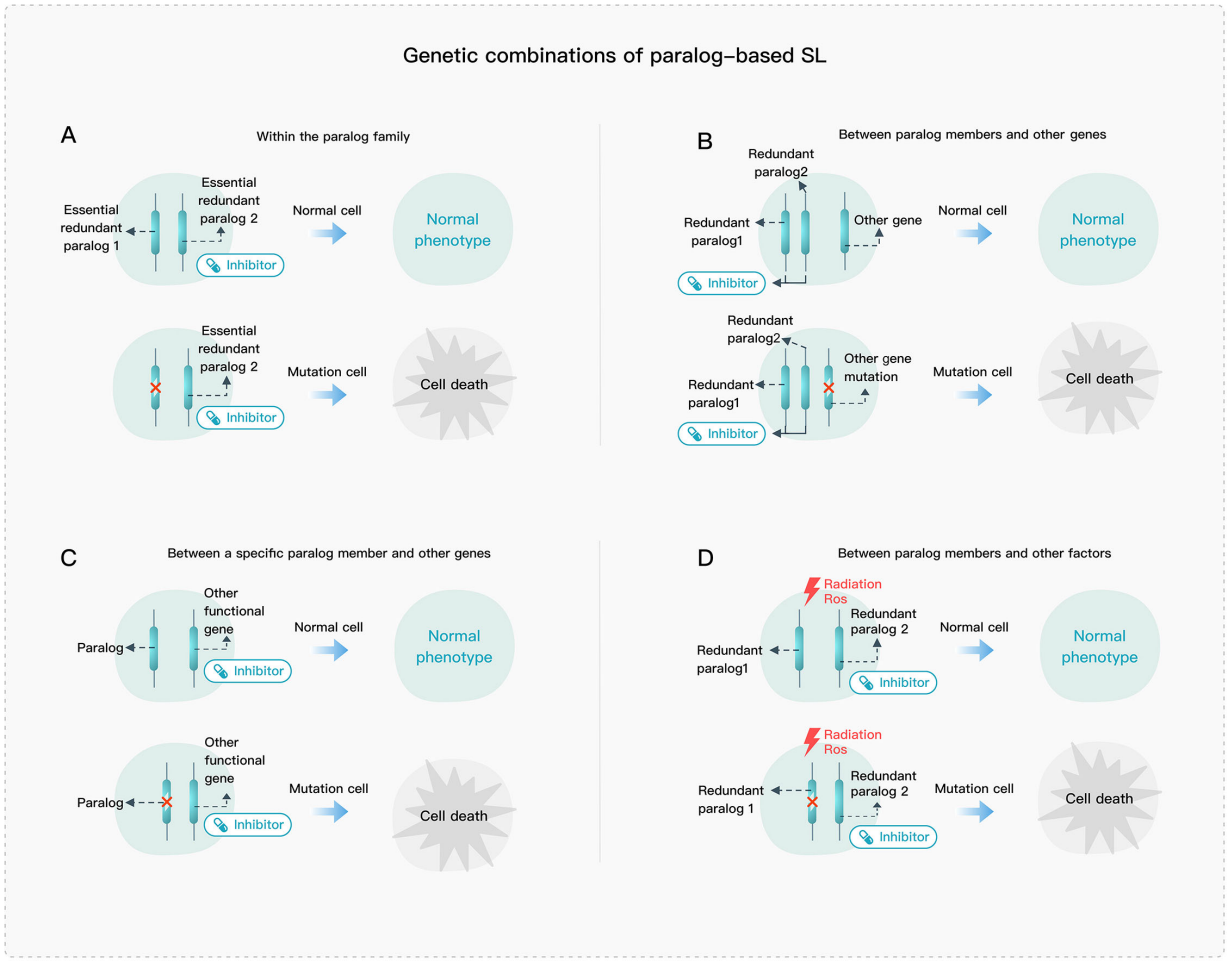
Genetic combination of paralog-based SL. **(A)** SL interactions within the paralog family. **(B)** SL interactions between paralog members and other genes. **(C)** SL interactions between a specific paralog member and other genes. **(D)** SL interactions between paralog members and other factors.

**Figure 3:**
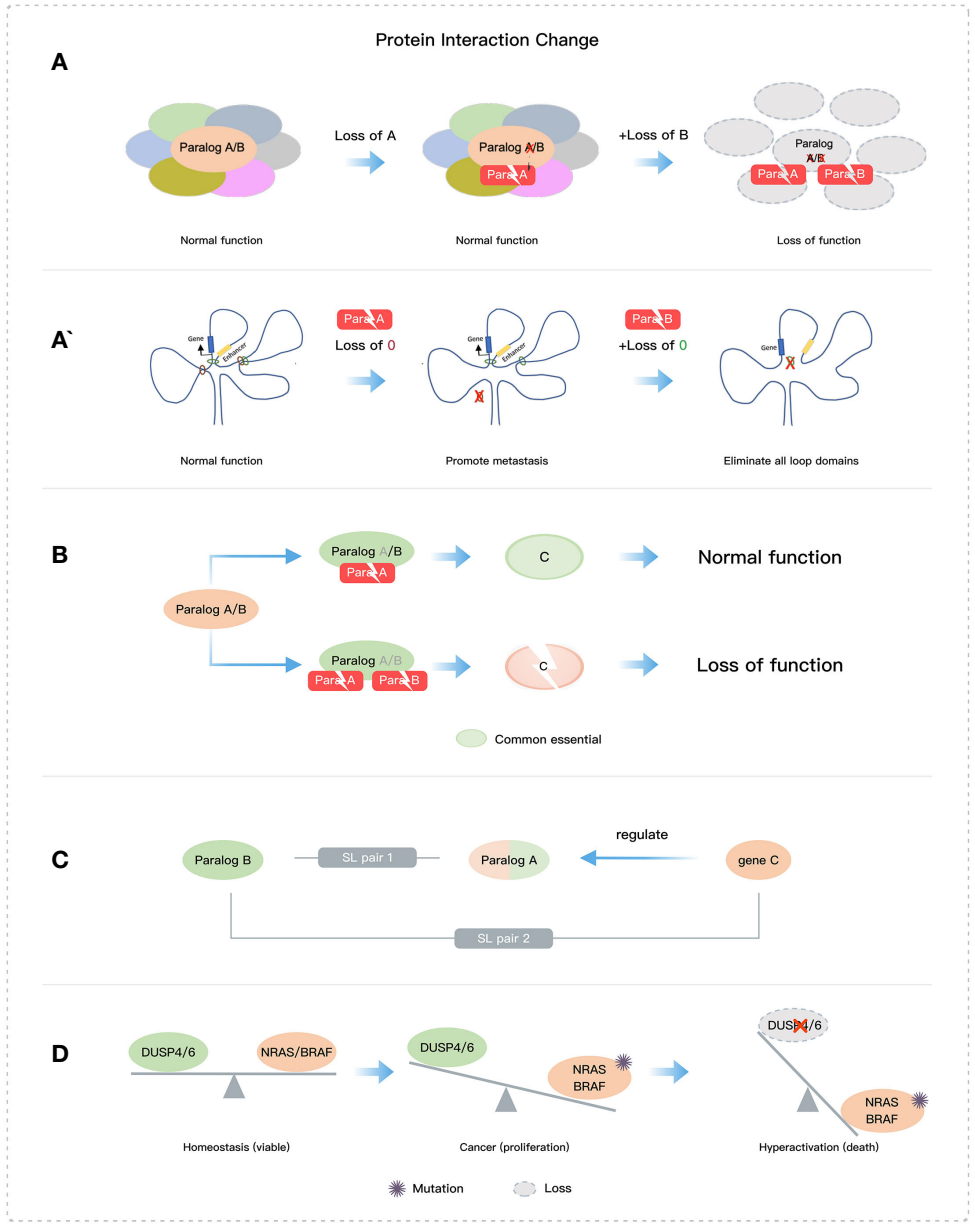
Some mechanisms of paralog-based SL. **(A)** Loss of core structures. **(B)** Loss of essential protein function. **(C)** The SL interaction of gene and its paralog or the regulator of its paralog. **(D)** Imbalance of two functions.

Paralog-based SL can generally be divided into four types: 1) SL interactions within the paralog family (**Figure 2A**); 2) SL interactions between paralog members and other genes (**Figure 2B**); 3) SL interactions between a specific paralog member and other genes (**Figure 2C**); and 4) SL interactions between paralog members and other factors (**Figure 2D**). The first type typically involves paralog members that play essential roles in a pathway or complex, such as the VPS4A and VPS4B ATPases in ESCRT. The second type involves genes that may exhibit complementary roles such as the BRCA and PARP genes. The third type can be further divided into two categories, one where SL interaction is formed between a gene and its paralog upstream regulatory genes, and the other where it is between functionally non-overlapping parts of paralogs and a functionally compensating gene. The fourth type is similar to the second one, but the targets of drugs and the cellular injury are often not specific, so the relationship cannot be specifically determined.

### 4.1 SL interactions within paralog families

#### 4.1.1 “Destabilization” of the core complexes

The loss of paralog members involved in a core protein complex can lead to critical dysfunction and cellular damage (**Figure 3A**). Mechanistically, protein complex structures and stoichiometry will be abnormal once there is a complete absence of mutually exclusive paralog members, which then triggers post-translational regulation of other members of the protein complex (94, 95). For example, in the SWI/SNF complex, the double deletion of ARID1A and ARID1B leads to the structural disruption of the complex (19), and when both SMARCC1 and SMARCC2 are deleted, the complex is almost completely destabilized (43, 96). Additionally, targeting HDAC1/2 in the NuRD complex, can lead to the selective degradation of essential subunits and impair transcriptional control (26). Another mechanism of SL may arise from altered chromatin interactions, as seen with the single deletion of STAG1 and STAG2, which can alter the distribution of cohesin complexes and cause changes in DNA-DNA loop formation and chromatin accessibility and interactions (**Figure 3A**’) (29, 97–99).

#### 4.1.2 Core protein abnormalities

Disrupting the stability and/or function of essential proteins by deleting paralogs is an effective mechanism for SL (**Figures 2A** and **3B)** (26). An example of paralog-based SL pairs with this mechanism is NXT1-NXT2, which regulates the stability of the essential protein NXF1. NXT1 or NXT2 forms heterodimers with NXF1 and constitutes the main mechanism for mRNA nuclear export. When both are absent, NXF1 is destabilized and rapidly degraded. The absence of NXF1 causes dysregulation of mRNA export from the nucleus to the cytoplasm and leads to abnormal cell growth or death (39, 100). Additionally, the exogenous expression of NXF1 can effectively restore mRNA export, and its protein stability is directly influenced by the presence of NXT1 or NXT2 (39, 101). Moreover, the deletion of both VRK1 and VRK2 reduces the essential protein BAF activity, leading to abnormal nuclear morphology, G2/M phase arrest, DNA damage, and eventually apoptosis (33, 34). The mechanism underlying the SL pair CREBBP and EP300 involves the abrogation of MYC expression (23, 24).

### 4.2 SL interactions between paralogs and other genes

The functional redundancy of paralogs hides some key SL targets, which highlights the existence of SL interactions between multiple paralog members and other genes (**Figure 2B**). BRCA1/2 proteins play a crucial role in DSB repair mediated by the homologous recombination (HR) pathway and have an indispensable contribution to maintaining genomic stability (63, 102). The PARP family has 17 members, among which PARP1 and PARP2 function as DNA damage sensing and transducing enzymes. PARP1 regulates cell proliferation and differentiation by repairing DNA single-strand break and DSB involved in the HR pathway, nucleotide excision repair, and base excision repair (10, 63, 103). PARP1/2 inhibitors are used as an SL therapy for BRCA-mutated cancers, although PARP1 and PARP2 can compensate for the deletion of each other in DNA repair. In the future, the SL relationships involving paralogs between compensatory pathways still need to be further clarified. Additionally, cells with NRAS or BRAF mutations are selectively impaired by the dual inactivation of DUSP4 and DUSP6 due to hyperactivation of the MAPK signaling pathway (18) (**Figure 3D**). This SL mechanism is not entirely dependent on paralogs but rather involves the balance of two functions (phosphorylation and dephosphorylation). Moreover, the SWI/SNF complex and the PRC2 complex containing EZH2 methyltransferase have been shown antagonistic activity in gene transcription (104, 105), of which EZH2 has an obvious anti-tumor effect on cell lines and xenografts with concurrent loss of SMARCA2 and SMARCA4 (106, 107). Dual loss of SMARCA4 and SMARCA2 also impacts tumor cell growth in PAX3:FOXO1+ARMS (108).

### 4.3 SL interaction between paralog single member and other genes/factors

Despite the redundancy of most paralog members, paralogs can acquire some non-overlapping functions through sub-functionalization and neo-functionalization. This can also lead to SL interactions with other genes (**Figure 2C**). For example, PRMT5 and PRMT9 are both type II arginine methyltransferases (109), but PRMT5 has a wider range of no redundancy functions compared with PRMT9 (110) and is a perceived SL target that can selectively kill tumor cells with MTAP deficiency (111). Similarly, despite the functional redundancy between SREBF1 and SREBF2, it was observed that SREBF2 had a significant negative genetic interaction with FASN while SREBF1 did not, indicating that they may have different functions that do not overlap (45). Also, CDK1 rather than CDK4/6 or CDK2 is selectively lethal in MYC-dependent cancers (112).

Genes can also form an SL interaction with upstream regulatory genes of their paralogs (**Figure 3C**). For example, BET inhibitors targeting BRD2 can downregulate the ARID1B expression, which indirectly triggers the SL interaction between ARID1A and ARID1B (113). Additionally, paralogs can also have SL effects in conjunction with other factors (**Figure 2D**), such as STAG2 mutations and DSB repair genes which increase sensitivity to cytotoxic chemotherapeutics and PARP or ATR inhibitors (27, 64).

## 5 Mechanism and influencing factors of paralog regulation

**Figure 4:**
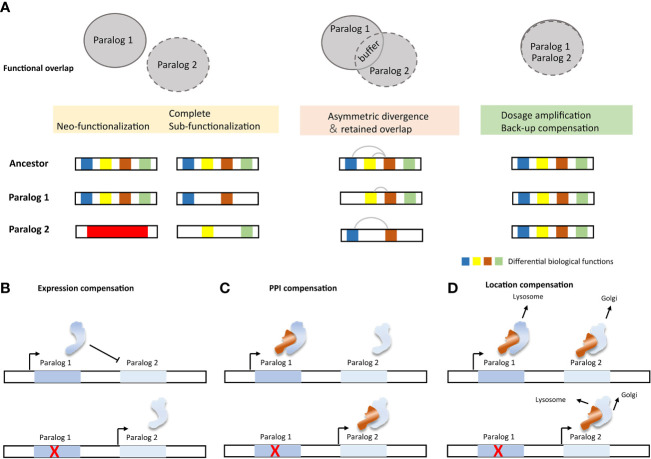
Impact factors and molecular mechanisms of paralog compensation. **(A)** Functional divergence and compensation capabilities of paralogs. **(B)** Active paralogous compensation forms (117).

Notably, not all paralogs are capable of functionally compensating. Functional divergence is a major limiting factor for their ability to compensate for their function (**Figure 4A**). The neo-functionalization suggests that the duplicate gene has developed a new or more specialized function, and the complete sub-functionalization of paralog genes suggests that the duplicate genes have completely split their functions from those of the ancestral gene. Therefore, the aforementioned two cases have the greatest theoretical functional divergence, and there is almost no functional compensation relationship among the paralogs in these cases. In contrast, the structural and functional entanglement in sub-functionalization often have partial functional overlap, that is, there is partial functional compensation (114). In addition to the partitioning of different biochemical functions, sub-functionalization also includes the partition of its expression and dosage sub-functionalization (1). Back-up compensation and dosage amplification involve fewer functional divergences, and the paralogs from this evolutionary route often obtain a selective advantage (115, 116). Theoretically, this case can provide complete compensation for missing members of paralogs.

The buffering effect of paralog can be subdivided into different mechanisms, passive paralogous compensation (analogous to “haplosufficient”) and active compensation (**Figure 4B**). Active compensation includes expression compensation, protein-protein interaction (PPI) compensation, and location compensation (117). Change in expression is the most easily monitored compensation method. However, the mode of action after up-regulation of expression still needs further confirmation. Paralogs involved in PPI are more likely to be subject to post-translational regulation (95). Compensation from PPI therefore often requires quantification of protein product rather than merely detection of changes in expression. Despite the limited reports on the regulation of SL paralog targets, evidence still suggests that multiple levels of regulation exist. For example, cancer cell lines with low UBB expression show elevated UBC levels, and inhibiting UBC induces UBB expression (118). In cells lacking Cohesin-SA2, the protein level of cohesin-SA1 increases, which changes the composition of the cohesin complex (29). Inactivation of DUSP4 leads to upregulation of DUSP6 in melanoma cells, likely through a transcriptional process (119). SMARCA4 inactivation leads to greater incorporation of the SMARCA2 subunit into the SWI/SNF complex (22).

From an alternate point of view, the paralog interaction relationship can be used as an argument for compensation ability. For example, the protective redundancy of paralogs partly depends on their independent functions. A large fraction of paralogous proteins may establish functional interdependence by heteromerization (physically interacting with each other), which reduces the ability of paralogous genes to compensate for each other’s loss (6). This indirect effect could stem from heteromeric paralogs having a larger number of PPI partners than non-heteromeric ones, thus loss-of-function (LOF) has a stronger effect on heteromeric ones and deal a greater degree of damage to the organism. Another research indicated that paralog pairs involved in protein complexes are more likely to show SL interactions (120). These findings are consistent with the SL interactions observed between mutually exclusive paralogs in the SWI/SNF complex (19, 22). In addition, from a dynamic evolution perspective, paralogs with earlier origins, and originating from whole-genome duplication are more likely to show SL interactions (6, 120).

## 6 Approaches for mining paralog-based SL pairs

Some SL pairs could be uncovered using genome-wide single gene perturbation (RNAi or CRISPR) combined with background abnormality in cancer cell lines (25, 26, 33, 37, 42, 45, 52). However, due to the functional redundancy, it is difficult to directly identify many essential paralogs for given cells. Instead, computational methods and systematic analysis across multiple cell lines will be feasible. Moreover, combinatorial screening methods are efficient at uncovering a more comprehensive set of paralog-based SL interactions. In addition, the mechanism of paralog-based SL interactions with other genes (**Figure 2B**) suggests that SL often involves not only two paralogs but also additional context-dependent genes. In this case, combinatorial paralog screening will be more effective. Therefore, experimental and computational approaches are complementary to each other.

### 6.1 Experimental approaches

To identify numerous and reliable paralog-based SL pairs, researchers have utilized combinatorial screening approaches (18, 44, 60, 77, 83, 121). However, due to the vast library size, they have to filter the paralogs based on various criteria such as sequence identity (60, 77, 83), paralog family size (44, 77), a single common orthologue in either *Caenorhabditis elegans* or *Drosophila melanogaster* (77), expression (60), gene essentiality (60, 77), and enzymatic paralogous genes (18). Several combinatorial screening tools are available in the paralog field and are continuously evolving. For instance, Parrish et al. and Thompson et al. utilized the relatively traditional *Streptococcus pyogenes* Cas9 enzyme system and focused on their interested paralog combinations (77, 83). To avoid the interference between the single-guide RNAs (sgRNAs) and increase the efficiency of combinatorial screening, Najm et al. expanded *Streptococcus pyogenes* Cas9 to orthogonal Cas9 enzymes from *Staphylococcus aureus* and *Streptococcus pyogenes* (75). Dede et al. took advantage of CRISPR/enCas12a to synthesize specific guide pairs in a single oligo and applied the enCas12a multiplex knockout system to identify paralog-based SL pairs (60). Gonatopoulos-Pournatzis et al. developed a hybrid Cas9-Cas12a enzyme from *Streptococcus pyogenes* and *Lachnospiraceae bacterium* to further improve efficiency (44). Many paralog-based SL interactions uncovered by these combinatorial screen approaches, and some new paralog-based SL pairs such as FAM50A-FAM50B (60, 77) and DUSP4-DUSP6 (18) have been confirmed in low-throughput experiments.

### 6.2 Computational approaches

The large-scale loss-of-function screening database the Cancer Dependency Map (DepMap portal, https://depmap.org/portal/) offers abundant single gene perturbation resources. Bioinformatic approaches leveraged these resources to systematically discover cancer-relevant paralog-based SL interactions by correlating genetic biomarkers (gene expression, copy number, mutation, and promoter methylation) with gene dependency (42, 60, 62, 118). For instance, this approach has uncovered paralog-based SL interactions such as UBB-UBC (118), MAGOH-MAGOHB (62), RPP25-RPP25, and DNAJC15-DNAJC19 (42). Moreover, an SL prediction model specifically for paralogs has been developed (49). The authors developed a random forest classifier to predict the robust SL interaction between paralogs by utilizing context-specific paralog SL interactions and 22 features reliable at evolutionary and mechanistic levels. ASF1A-ASF1B and COPS7A-COPS7B were the highly-ranked predicted SL pairs and were further validated by RNAi (49). As more cell lines undergo combinatorial perturbation of paralog pairs, the number of ground-truth positive and negative labels will increase, offering more opportunities for methods such as Network-based and Deep learning methods. Details of these methods can be found in reference in the review papers by Wang et al. (122) and Tang et al. (123).

## 7 Conclusion and future perspectives

The concept of SL has provided robust and novel strategies for precision cancer therapies. However, the compensatory relationship of functionally redundant genes may obscure potential therapeutic utility. Functional buffers, such as paralogs, are key factors in background dependence and compensation. Here, we reviewed the functions of paralogs in terms of SL interaction, the four genetic combination patterns of paralog-based SL interactions, the mechanisms and the factors affecting the buffering effect of paralogs, and efficient mining methods for SL paralog pairs. It provided a theoretical foundation for the mining of more paralog-based SL targets.

The evolutionary characteristics of tumor initiation and development are closely tied to the robustness provided by buffer systems. Paralog variations in tumor cells provide rich resources for developing SL-based cancer therapies. Thorough characterization of the paralog buffer system can be useful for future tumor research and therapeutic development. This includes understanding the importance of paralog members, predicting functional divergence and buffering effects, and identifying regulatory differences among paralog members. The same paralog SL gene pair can elicit different responses in different cell lines (24) depending on factors such as the deletion of a background gene, the existence of different compensation pathways, and the presence of other non-paralog compensatory genes. Achreja et al. had attempted to identify collateral lethal genes through collateral lethal gene identification *via* metabolic fluxes (CLIM), opening up a new avenue for finding compensatory genes beyond paralog genes (69, 124). The possible functional divergence of paralog could also be explored for tumor heterogeneity characterization (125–128).

In conclusion, paralog-based SL provides a powerful genetic engine for cancer research and clinical development. Understanding the mechanisms of SL pairs and the characterization of buffer systems in tumors will shed light on future research and development of precision cancer therapy.

## Author contributions

YX and YZ initiated the project. XY wrote the manuscript. YZ provided feedback on the manuscript. All authors contributed to the article and approved the submitted version.
